# Supplementary material for: Altered Grey Matter-Brain Healthcare Quotient: Interventions of Olfactory Training and Learning of Neuroplasticity
Source: Life (Basel). 2023 Feb 28;13(3):667. doi: 10.3390/life13030667 (PMC10052964; doi:10.3390/life13030667)
Supplement: Supplementary file 1 [file life-13-00667-s001.zip › life-2194319-supplementary.pdf]

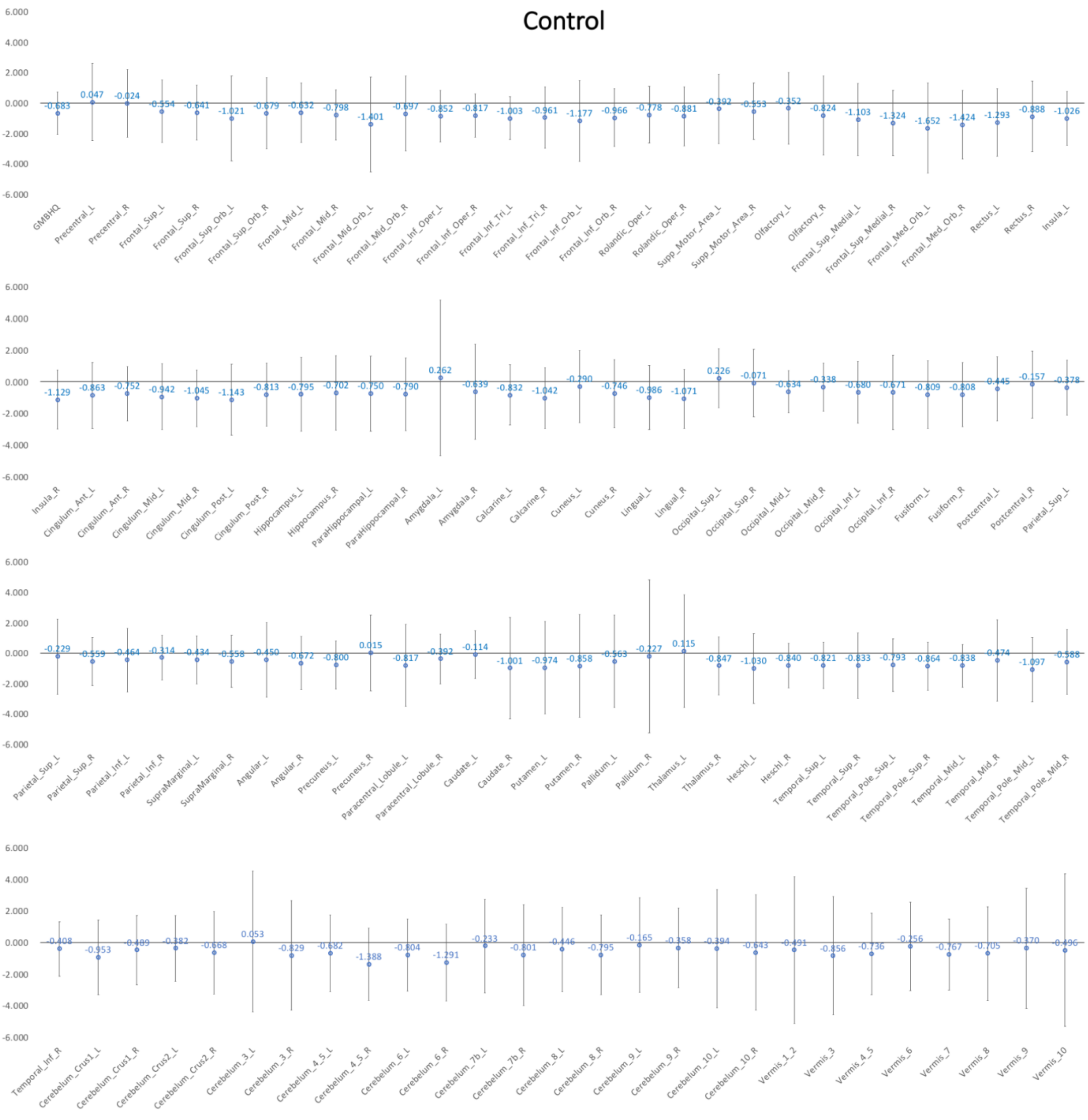

Supplemental Figure S1. Brain healthcare quotient changes of each brain region

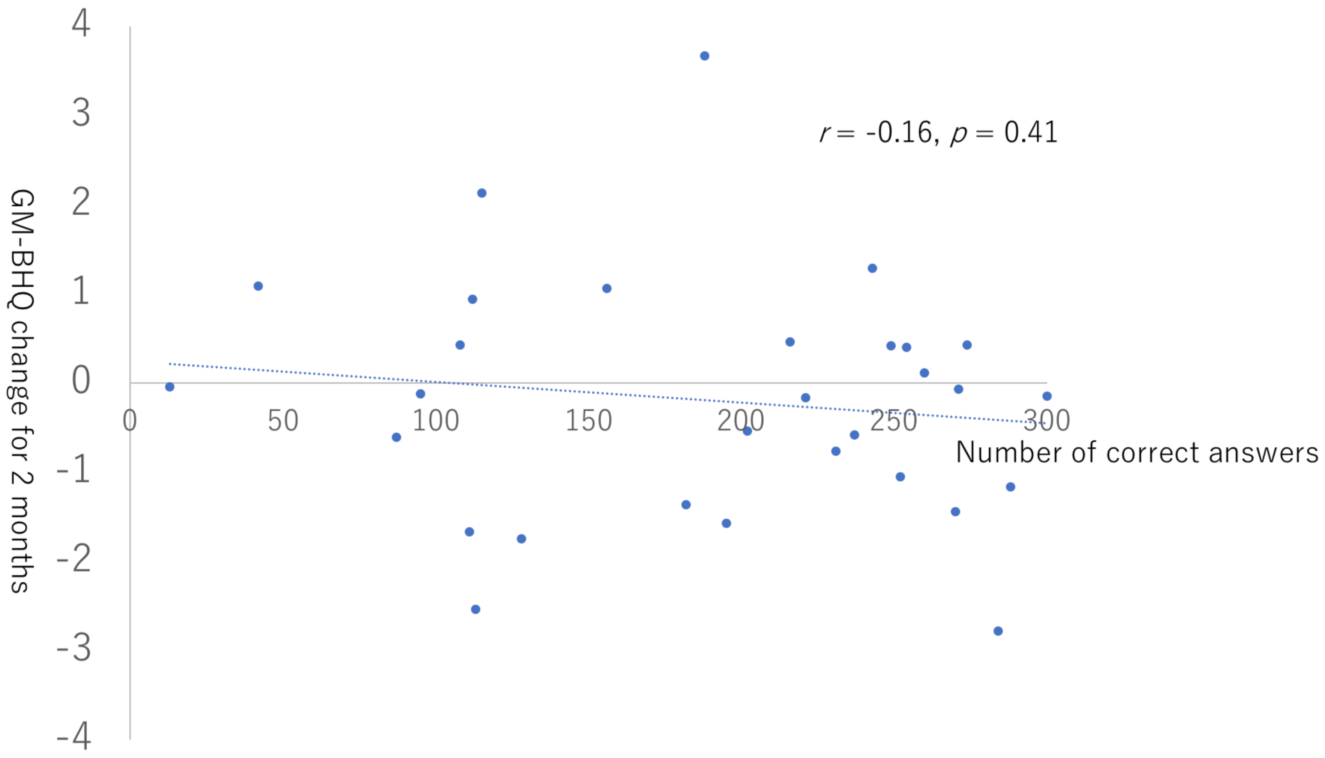

Supplemental Figure S2. Relationship between grey matter-brain healthcare quotient changes and scores of olfactory training.

Supplementary Data

Supplementary Data 1. The questionnaire on neuroplasticity used to briefly test participants' knowledge after watching the video (true-false test)

1. After childhood, the brain will not change even with training.
2. Changes in the brain structure are responsible for long-term improvement in motor skills.
3. One of the key factors that cause changes in the brain structure is the person's own behaviour.
4. There are no major individual differences in the way we learn or in the training for changes in the brain structure.
5. In the process of practicing, as the difficulty increases and more effort is required, more learning occurs in the brain, and more structural changes occur.

Supplementary Data 2. Twenty-eight brain healthcare messages.

6. Even in adulthood, the brain continues its propensity to change. Therefore, continuous training is needed.
7. The more difficult a task is and the more effort it requires, the more likely that changes in brain function and structure will occur.
8. It has been found that when faced with a difficult task, people who believe that their abilities will change are more likely to experience changes in brain function than those who believe that their abilities will remain the same.
9. It has been shown that not only physical training but also imagery training, where one visualizes one's body moving, can improve skills and change brain structure.
10. It has been revealed that when we experience something, it is easier for the brain structure associated with that experience to change if we pay attention to it, rather than just going through the experience randomly.
11. It has been found that people who get good sleep have larger hippocampi, which are involved in memory and emotional control.
12. Drinking alcohol leads to brain atrophy, and even moderate amounts of alcohol are thought to cause brain atrophy. Conversely, it has been found that brain atrophy improves in heavy

drinkers when they limit their drinking. Drinking small amounts of alcohol is expected to improve blood circulation and prevent vascular diseases and dementia.

13. At the research level, nicotine has been reported to have neuroprotective effects, but it is clearly shown that tobacco damages the brain and leads to brain atrophy.
14. Social interaction is very important for brain health. It has been found that people who have more social interaction have a larger prefrontal cortex, which is involved in thinking and decision-making abilities.
15. We know that the longer we are educated in school, the bigger our brains become. Education has also been reported to help prevent age-related brain atrophy. Even as we get older, learning something has a positive effect on our brain.
16. Brain atrophy is common in aging, but aerobic exercise such as walking has been shown to reduce or improve brain atrophy.
17. It has been found that lifestyle-related diseases such as diabetes and hypertension cause brain atrophy. Maintaining a healthy lifestyle is important for brain health.
18. There is a close relationship between obesity and brain atrophy, and it has been suggested that eliminating obesity may improve brain atrophy.
19. Diets such as calorie restriction for the purpose of maintaining health and proper weight have been shown to affect not only weight loss but also improved mood, quality of life, and even cognitive function. It has been shown that such diets can affect brain function and structure.
20. During stress, the body secretes a hormone called cortisol to protect itself from stress; however, excess cortisol can damage the brain, causing atrophy of the hippocampus and frontal lobe. Stress management is important for brain health.
21. Appropriate exercise and a healthy diet have been shown to have a preventive effect on dementia and improve cognitive function. Dementia is a disease of the elderly, but to prevent dementia, it is important to maintain a healthy lifestyle from a young age, such as in your 30s and 40s.
22. It has been reported that a well-balanced diet with lots of vegetables and moderate amounts of fish can reduce age-related changes in the brain.
23. The unsaturated fatty acids found in fish, such as DHA, are good for brain health and can help prevent dementia, depression, and myocardial infarction. Studies have also shown that an active DHA intake for 6 months resulted in increased brain activity on magnetic resonance imaging.
24. Vitamins are important for brain health, with vitamin B being particularly important. It is hoped that an intake of vitamin B will help reduce brain aging.
25. Playing a musical instrument requires the integrated use of functions that span a variety of brain areas, including not only the motor area for playing but also the visual area for looking at the score and instrument, the auditory area for listening to the sound being played, the memory area for memorizing the score, and the higher-order cognitive function area for integrating these areas. Therefore, it has been found that various brain areas can be expanded through continuous training.
26. Daily yoga and mindfulness meditation have been shown to expand the hippocampus, which is involved in memory and emotional control.
27. Dance requires the perception of sound and the integration of rhythmic movements of the arms and legs. It has been shown that various brain areas can be expanded by continuing such dancing daily.
28. It has been found that the anterior insular cortex, which is involved in empathic understanding of the physical sensations and emotions of others, expands through continuous compassionate meditation for the happiness of all living beings.
29. An interesting study found that people who were exposed to more of nature in their lives had multiple brain regions that were expanded.
30. Air pollution has been shown to cause a variety of diseases and to have a significant impact on cognitive function. Additionally, it can worsen the function and structure of the brain.
31. The key to neuroplasticity is to do training and imagery that involves experience, to do it continuously, and to do it wholeheartedly.
32. The best methods, training, lifestyle, and environment for changing the structure of the brain vary greatly from person to person. Therefore, it is very important to find a method that works for you.

33. Even in adulthood, the brain continues its propensity to change. As we have seen, there are many factors that contribute to this change, including lifestyle, eating style, exercise, learning, social life, and one's environment. For each factor, try to find the best drop-off point for you. This message will end today. Thank you for all your hard work over the years.

Supplementary Data 3. Questionnaire for behaviour changes.

In the 2 months since your last MRI scan, we would like to ask you about your new attempt in your daily life.

Choose the answer that most applies to each question.

1. Not at all
2. A little applicable
3. Applies to some extent
4. Quite applicable

(Scores from 0: not at all to 3: Quite applicable)

1. I visited various internet sites that I had never visited before
2. I went to shops and places that I've never been to
3. Participated in events (including online) that I have never attended before
4. I started some play, hobbies and lessons that I've never done before
5. Worked on quitting lifestyle-related habits that I thought were not good for me
6. Worked on starting a lifestyle that I thought was good for me
7. I read some self-development books
8. I spend more time tackling challenges than ever before
9. Worked on various new things
10. I heard some music I've never heard before
